# A Novel Posture for Better Differentiation Between Parkinson's Tremor and Essential Tremor

**DOI:** 10.3389/fnins.2018.00317

**Published:** 2018-05-17

**Authors:** Bin Zhang, Feifei Huang, Jun Liu, Dingguo Zhang

**Affiliations:** ^1^State Key Laboratory of Mechanical Systems and Vibrations, Robotics Institute, Shanghai Jiao Tong University, Shanghai, China; ^2^Department of Neurology, Rui Jin Hospital, School of Medicine, Shanghai Jiao Tong University, Shanghai, China

**Keywords:** Parkinson's disease, essential tremor, tremor differentiation, postural position, arm-rested posture

## Abstract

Due to a lack of reliable non-invasive bio-markers, misdiagnosis between Parkinson's disease and essential tremor is common. Although some assistive engineering approaches have been proposed, little acceptance has been obtained for these methods lack well-studied mechanisms and involve operator-dependent procedures. Aiming at a better differentiation between the two tremor causes, we present a novel posture, termed arm-rested posture, to ameliorate the quality of recorded tremor sequences. To investigate its efficacy, the posture was compared with another common posture, called arm-stretching posture, in fundamental aspects of tremor intensity and dominant frequency. A tremor-affected cohort comprising 50 subjects (PD = 26, ET = 24) with inhomogeneous tremor manifestation were recruited. From each subject, acceleration data of 5 min in terms of each posture were recorded. In the overall process, no operator-dependent procedures, such as data screening, was employed. The differentiation performance of the two postures were assessed by the index of discrimination coefficient and a receiver operating characteristic analysis based on binary logistic regression. The results of the differentiation assessment consistently demonstrate a better performance with the arm-rested posture than with the arm-stretching posture. As a by-product, factors of disease stage (incipient, progressed stage), spectrum estimate (PSD, bispectrum) and recording length (5–300s) were investigated. The significant effect of disease stage was only found in PD in terms of tremor intensity [*F*_(1, 516)_ = 7.781, *P* < 0.05]. The bispectrum estimate was found to have better performance than the PSD estimate in extracting dominant frequency in terms of the discrimination coefficient. By extending the recording length, we noticed an increase in the performance of dominant frequency. The best result of the arm-rested posture was obtained with the maximum recording length of 300 s (area under the curve: 0.944, sensitivity: 92%, 1-specificity: 0%, accuracy: 96%), which is better than that of the arm-stretching posture in the same condition (area under the curve: 0.734, sensitivity: 54%, 1-specificity: 12%, accuracy: 72%). Thus, we conclude that the arm-rested posture can assist in improving tremor differentiation between Parkinson's disease and essential tremor and may act as a universal tool to analyze tremor for both clinical and research purpose.

## 1. Introduction

Different from physiological tremor that accompanies normal body movements (Raethjen et al., 2000), pathological tremor impairs the flexibility and coordination of motor function by eliciting large-amplitude involuntary muscle oscillation activities (Rocon et al., 2004; Helmich et al., 2013). Among its 11 confirmed etiologies (Deuschl et al., 1998), Parkinson's disease (PD) and essential tremor (ET) account for up to 90% of the tremor-affected population (Deuschl et al., 1998; Thanvi et al., 2006). However, even between these two most common causes, there is a high rate of misdiagnosis. Approximately 25% of the PD patients are misdiagnosed as ET cases (Rizzo et al., 2016). The optimal medical treatment of these PD patients is therefore delayed. The best opportunity of controlling the irreversible progress of the disease is missed (Mark, 2007).

Resting tremor that occurs in a fully supported arm serves as an important symptom in differentiating PD from ET in clinical diagnosis (Jankovic, 2008). However, its existence does not fully corroborate the diagnosis of PD because it exists in approximately 20% of ET cases (Elble and Koller, 1990; Cohen et al., 2003) and meanwhile 25% Parkinsonian cases lack resting tremor (Helmich et al., 2013). In addition, resting tremor may not occur until years after onset (Schneider et al., 2007). Although some gold standards, such as the UK PDS Brain Bank Criteria, have been published (Hughes et al., 1992), the misdiagnosis rate between PD and ET has not been reduced drastically. This may be due to the fact that the clear diagnosis of PD requires a series of relevant symptoms based on the UKPDS Brain Bank Criteria, which takes a long period, normally no less than 5 years, to confirm (Schneider et al., 2007). Although there are some nuclear imaging techniques, such as, DaTSCAN or DOPA PET (Pavese et al., 2011), high cost issues and lack of sophisticated practice greatly restrict their application in clinic settings. Therefore, efficient and easy-to-access methods to facilitate tremor diagnosis are still in great demand.

Meanwhile, researchers have been seeking to better differentiate PD and ET from the engineering perspective. Many statistical features from kinetic signals of tremor, including tremor intensity, dominant frequency, bursting duration (Milanov, 2001), shape factors (Deuschl et al., 1995), tremor stability index (Di Biase et al., 2017) etc., have been discovered to be statistically different between PD and ET. However, due to overlaps in feature distribution, none of these features alone are able to separate the two tremors (Capildeo and Findley, 1984; Calzetti et al., 1987; Milanov, 2001). Thus far, some workable methods has been published. The majority of them utilize the kinetic measurement of tremor acceleration or electromyographic (EMG) signals that are easy to access and are based on a series of complex extracted features and methodologies of machine learning. Centering on the high non-Gaussianity and non-linearity of tremor acceleration, Jakubowski et al. (2002) introduced high-order statistics (HOS) to tremor differentiation and achieved a satisfactory accuracy rate of 97% by applying the method of neural network in separating three categories of tremor, including PT, PD, and ET (Jakubowski et al., 2002). His study was followed by Ai et al. (2007), who simplified the method by greatly reducing the number of high-order statistical features (Ai et al., 2007). Engin et al. (2007) combined some other complex mathematical features, such as the wavelet transform based entropy feature etc., for tremor differentiation with EMG signals (Engin et al., 2007). Ai et al. (2011) applied the empirical mode decomposition in separating ET and PD and obtained some remarkable results (Ai et al., 2011). In recent years, the approach of data mining based on a huge amount of data (Palmes et al., 2010) or of features (Povalej Bržan et al., 2017) has been used to investigate this issue.

In spite of the remarkable results achieved, few of these methods has been widely used in clinical practice. Thus far, clinical diagnoses of tremor still largely depends on the doctors' experience. We attribute this fact to the shortcomings of the published methods including: (1) the setup of the method targets mainly patients who satisfy specific requirements, such as with moderate-to-severe tremor; (2) the process of data processing requires data screening for data segments that exhibit obvious and stable characteristics (Jakubowski et al., 2002), thus, it might be operator-dependent and require certain relevant experience in it. (3) the method is without well-studied mechanism and lack of convincing physiological explanation. From feature extraction to category prediction, the overall process may be regarded to be inexplicable.

**Figure 1:**
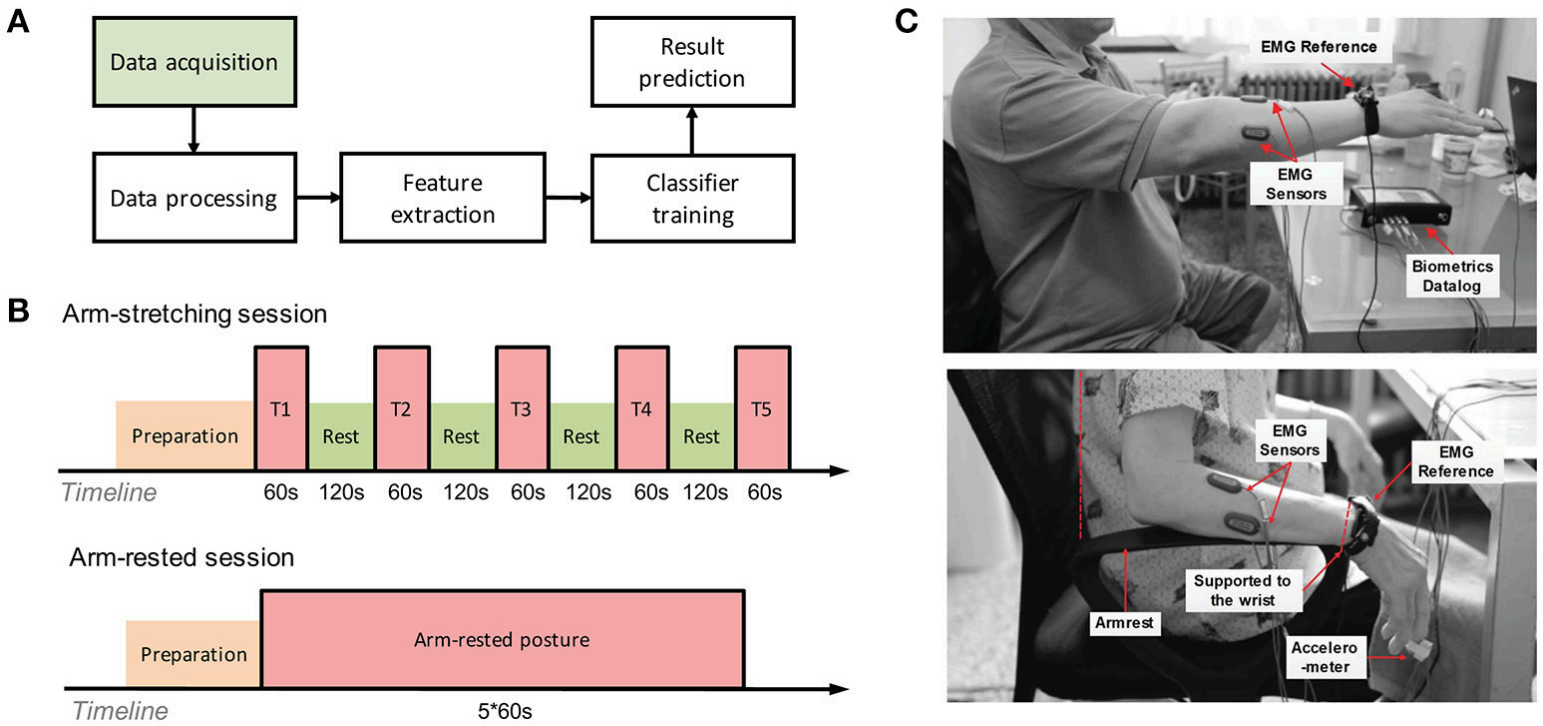
Experimental setup. **(A)** Example of a general machine learning workflow for tremor differentiation. **(B)** Experiment protocol incorporating two sessions for each subject. Each session involved one of the postures concerned, i.e., the traditional arm-stretching posture (P1) and the arm-rested posture (P2) proposed in this research. **(C)** The lateral view of both postures. In the arm-stretching posture (up), the seated subject was required to stretch out one of his/her affected arms and to hold the posture. In the arm-rested posture (down), the subject was seated with both arms supported at the wrists. The height of the seat and the position of body were adjusted to make subject's forearm muscles passively strained.

Central to these problems is the complex, fluctuant nature of tremor that makes data screening and complex methods necessary (Bain, 1998). However, in the abovementioned engineering methods, most efforts were made to the procedures after they acquired tremor data (Figure 1A). In terms of postural context, they simply chose the arm-stretching context, corresponding to the arm-stretching posture, to consistently elicit tremor in both PD and ET patients (Deuschl et al., 1995; Ai et al., 2007; Engin et al., 2007). Since tremor has been reported to relate to a quantity of factors, including the physical and mental state of patient and even environmental parameters, such as environment temperature (Bain, 1998), ignoring the process of obtaining tremor data may add to the problem of fluctuant, unstable tremor nature.

This potential oversight in the current research prompted us to start the research of investigating whether it is possible to reduce the difficulty of tremor classification by ameliorating the process of tremor recording, such as the postural context. Not many studies have focused on this aspect on tremor differentiation. Uchida et al. (2011) compared the 4 most common postural contexts (stretching arm, resting, writing and walking) and demonstrated the significant effect of postural context over basic tremor properties (Uchida et al., 2011). Burne et al. (2004) investigated different response in postural tremor with muscle loading and found muscle loading a potential factor in isolating PD and ET (Burne et al., 2004). Farkas et al. (2006) used a pen-like tube to record a combination of resting and postural tremor under a writing context, and found a significant difference in bilateral asymmetry of tremor intensity between PD and ET (Farkas et al., 2006). Papengut et al. (2013) validated the suppression response of rest tremor amplitude in raising arms (Papengut et al., 2013). However, none of the above studies provide a complete separation between PD and ET.

In this research, we aim to improve tremor differentiation between PD and ET by ameliorating the quality of raw tremor data. A novel postural position, termed arm-rested posture, is designed by removing redundant freedoms of muscles and joints during measurement. To avoid the loss of generality, we compare it against the posture called arm-stretching posture, which is the most common posture in other research to elicit postural tremor. The effect of the two postures was evaluated in two fundamental aspects which are tremor intensity and tremor frequency. The hypothesis is that, while compared with arm-stretching posture, the proposed postural position yields better results in tremor differentiation. In addition, we also investigate the effect of the recording length, the disease progression stage and the spectrum types in tremor differentiation. Finally, we explore whether it is possible to give a complete separation between the PD cohort and the ET cohort without involving operator-dependent data-screening and inexplicable classifying procedures.

## 2. Methods

### 2.1. Subjects

Fifty adult patients in total participated in the experiment (Table 1). They were all regular outpatients recruited by the department of neurology of Rui Jin Hospital (Shanghai, China). Of all recruited PD subjects (*n* = 26), most had postural tremor (*n* = 21) and a large portion had resting tremor (*n* = 20). The modified Hoehn and Yahr (H&Y) stage of the overall PD cohort ranged from 1.0 to 3.0 [2.3 ± 0.9 (Mean ± SD)] while the UPDRS score ranged from 18 to 49 [28.5 ± 7.3 (Mean ± SD)]. Based on the calculation protocol by Tomlinson et al. (2010), the L-dopa-equivalent daily dose (LEDD) was found to range between 25 and 625 mg [247.1 ± 160.5 (Mean ± SD)]. All recruited ET patients (*n* = 24) had postural tremor (*n* = 24) and a small portion of them had resting tremor (*n* = 6). Before participation, all participants provided written consent after being given notice on the purpose and procedures of the experiment. This research was approved by the local Ethics Committee of Shanghai Jiao Tong University.

**Table 1:** Basic information and clinical features of the subjects.

	**PD1**	**PD2**	**ET1**	**ET2**	**PD**	**ET**	***P*-value**
Num of cases	13	13	12	12	26	24	–
Gender, M/F	4/9	5/8	2/10	5/7	9/17	7/17	0.767
Onset side, L/R	6/7	6/7	4/8	6/6	12/14	10/14	0.783
Age, yr (Mean ± SD)	68.8 ± 6.4	71.5 ± 6.0	62.6 ± 11.6	63.8 ± 13.0	70.2 ± 6.1	63.2 ± 12.1	0.645
Duration, yrs (Mean ± SD)	2.1 ± 1.5	14.4 ± 9.7	4.0 ± 2.6	21.4 ± 13.5	8.2 ± 9.3	12.7 ± 13.0	0.166
H&Y, score (Mean ± SD)	1.5 ± 0.6	3.0 ± 0.0	–	–	2.3 ± 0.9	–	–
UPDRS, score (Mean ± SD)	23.0 ± 3.6	33.9 ± 5.8	–	–	28.5 ± 7.3	–	–
LEDD, mg (Mean ± SD)	134.6 ± 101.3	359.6 ± 126.4	–	–	247.1 ± 160.5	–	–

*PD, Parkinson's disease; ET, essential tremor; H&Y, Hoehn and Yahr stage; UPDRS, Unified Parkinson's Disease Rating Scale; LEDD, L-dopa-equivalent daily dose*.

The criteria of recruitment for both the PD cohort and the ET cohort included: (1) between the ages of 30–80 years old; (2) with confirmed diagnosis of either PD or ET; (3) with upper limb tremor (at least one type of tremor, such as resting tremor, postural tremor and kinetic tremor); (4) particularly for PD patients, at modified H&Y stages 1–3 (Hoehn and Yahr, 1967). The diagnosis of PD was established on the UKPDS Brain Bank Criteria (Hughes et al., 1992) while that of ET was determined based on the consensus statement of movement disorder society on tremor, which requires taking patient's clinical history and results of general neurological examinations into an overall consideration (Deuschl et al., 1998). Moderate-to-severe tremor was not considered as a necessary factor in subject recruitment. The inhomogeneity of tremor manifestation served our aim to recruit cohorts in accordance with the vast majority of tremor patients.

Patients would be excluded if any of the following conditions were true: (1) a disease history of both PD and ET; (2) being treated with any neuromodulation therapy, including deep brain stimulation (DBS), transcranial direct current stimulation (tDCS), transcranial magnetic stimulation (TMS) etc., within recent two weeks; (3) mental issues, including anxiety, dementia, hallucination and delusion etc.,; (4) other condition that affect the subject in completing the overall experiment, such as cognitive disorder; (5) a strong reliance on medications relevant to PD or ET, such as anti-Parkinson medications. To exclude drug effects, subjects were told to discontinue anti-tremor medications before the day of experiment, which helped ensure a withdrawal period of more than 12 h. Subjects resumed medication immediately after the experiment ended.

Based on disease progression severity, all subjects in each cohort were labeled into two different stages, which were the incipient stage (S1) and the progressed stage (S2). In the PD cohort, subjects at the incipient stage (PD1) were defined as PD patients with a modified H&Y Stage 1–2.5 while those with a modified H&Y Stage 3 were labeled at the progressed stage (PD2). The rationale behind this was that the modified H&Y Stage 3 is regarded as the beginning stage of the end stages (Stage 3–5) while those lower stages (Stage 1–2.5) are treated as early stages of PD (Hoehn and Yahr, 1967). For ET, tremor is the unique symptom and generally progresses slowly over time. Thus, in the ET cohort, subjects were separated with disease duration. Subjects with an ET duration less than 5 years were labeled at the incipient stage (ET1) while those with a duration more than 5 years would be labeled at the progressed stage (ET2).

### 2.2. Procedures

The overall experiment was carried out in a specific room in the in-patient department of Rui Jin Hospital (Shanghai, China). The room temperature was kept at 26^*o*^*C* by an air-conditioning system throughout the experiment. Irrelevant personnel were removed from the room, and the door was closed to ensure the stability of the experiment environment.

Two kind of measurements, including acceleration measurement and surface EMG (sEMG) measurement, were applied in the experiment. They were both acquired with the help of a commercial device system called Biometrics Datalog (Biometrics Inc., USA). After the documentation of the subject's clinical history, his/her more affected arm was first determined by an experienced physician by visually checking which arm was more severely affected by the upper limb tremor. Particularly for PD patients, the more affected side was always in accordance with the onset side of PD. A 3-axis accelerometer was fixed onto the third knuckle of the middle finger on the more affected side. Four sEMG sensors were fixed onto the muscle bellies of the flexor carpi radialis (FCR), flexor carpi ulnaris (FCU), extensor carpi radialis (ECR) and extensor carpi ulnaris (ECU) on the more affected side. This was done according to the Non-Invasive Assessment of Muscles—European Community Project (SENIAM, the surface electromyography for the non-invasive assessment of muscles) recommendations for sEMG electrode placements (Hermens et al., 1999). All signals were digitized in 1000 Hz and transmitted to a PC through Bluetooth. Data were then converted and saved in special formatting.

Each subject in both of the cohorts underwent two sessions in the experiment (Figure 1B). The first session involved the traditional posture of the arm-stretching posture (P1), where the seated subject outstretched his/her more affected arm forward as shown in Figure 1C (up). The posture involved in this session was reported to be the most sensitive in tremor differentiation (Ai et al., 2007) and has been performed in many published studies (Milanov, 2001). In order to enhance reproducibility of the posture, we restricted the subject's postural position in the following aspects: (1) the wrist, the elbow and the shoulder of the more affected side should align in a horizontal line that is perpendicular to the plane of the trunk; (2) the more affected arm should be outstretched in full extension; (3) fingers should be closed with a flat hand and the palm facing downward. Five trials were incorporated in the session and between each there was a 1-min rest to avoid muscle fatigue after the continuous muscle contraction. Before each trial, there was a sound cue followed by a 5-s period to alert the subject to prepare for the posture.

In the other session, the subject performed the arm-rested posture (P2) that is proposed here. In this posture, the subject was required to sit upright with both arms rested and both palms facing downward [Figure 1C (down)]. Note that both arms were only supported at the wrists (by the armrests), rather than in a full support of forearm. We also adjusted the relative height of the armrests to the seat and the relative horizontal distance of the subject's trunk to the backrest to ensure the elbow hanging at a moderate distance (2–5 cm) above the plane of the armrest. The main purpose of these adjustments was to keep the subject's forearm muscles and tendons passively strained. During recording, both upper limbs of the subject should be fully relaxed, which were monitored in real time by checking the plotted sEMG signals of forearm muscles on screen. The arm-rested posture was kept continuously for 5 min (1 trial in total) until the end of recording.

For each subject, the overall time duration of the experiment was approximately 30 min. Subjects were told to concentrate on the instructions of the experiment and to avoid irrelevant behaviors throughout the experiment.

### 2.3. Signal processing and statistics

The methods of signal processing were implemented with Matlab (R2016a, MathWorks Inc., USA). Only the acceleration signals of the tremors were utilized for analysis while EMG signals were only utilized for real-time muscle activation monitoring in data recording. We neglected EMG data because they were found to lack inter-subject consistency in muscle activation pattern and good connectivity between muscle and sensor in some aged subjects. For ease of calculation, all data sequences of the tremor accelerations were first segmented by splitting the sequences every consecutive 30 s (non-overlap), which means the data sequence of 5 min would be split into 10 segments for each subject in each posture. Since in most cases the re-emergent latency of tremor is less than 10 s (Jankovic et al., 1999), each segment was assumed to have incorporated tremor components which characterized the disease. Denoising notch filters of 50 Hz and higher harmonics were then applied to eliminate the 50-Hz power line interference. Here in this research, the acceleration signals were supposed to characterize the kinetic features of movements that generally ranged below 20 Hz. Thus, in the consideration of removing zero shifting of hardware sampling and voluntary movement components that are normally below 1 Hz, we applied a second-order Butterworth bandpass filter with a passband of 1–20 Hz. The technique of zero-phase filtering was employed to avoid phase delay issues (Gustafsson, 1996). The filtered data were then down-sampled to 100 Hz for ease of calculation.

In characterizing tremor, we considered the two fundamental aspects, which were respectively tremor intensity and tremor frequency. The mean absolute value (MAV), which represents the algebraic mean of data sequence, was calculated from each filtered acceleration segment to feature tremor intensity (Equation 1).

(1) $$\begin{array}{r} MAV=\frac{1}{N}\overset{N}{\underset{k\;=\;1}{\sum }}|x\left(k\right)| \end{array}$$

where *MAV* denotes the mean absolute value of time sequence ${\left\{x\left(n\right)\right\}}_{n\;=\;0}^{N-1}$ with a finite amount *N*.

In featuring tremor frequency, we chose the feature of dominant frequency calculated from two different spectrum estimates: power spectrum density (PSD) estimate and bispectrum estimate. While the PSD estimate has achieved wide acceptance and many applications in the field of signal processing, bispectrum estimate is less known but still important in cases where a second-order statistical description is not sufficient. Based on previous studies, a tremor is a non-Gaussian random process with high non-linearity (Jakubowski et al., 2002) where high-order statistical analysis is necessary. We employed Welch's averaging periodogram method for the PSD estimate (Welch, 1967; Schuster, 2012) (Equation 2) and the Fast Fourier transform (FFT) based direct approach for bispectrum estimate(Collis et al., 1998) (Equation 3). In the PSD estimate, a sliding Hanning window was used to average over short periodograms and stabilize the PSD results. The dominant frequency of the PSD estimate was defined by calculating the frequency corresponding to the maximum power value on the power spectrum. In the bispectrum estimate, we applied the smoothing approach of multiplying the third-order cumulant *C*(*k, l*) with a sliding Hanning window to stabilize the bispectrum estimate. The diagonal slice of the bispectrum estimate was calculated as having ω_1_ = ω_2_ in Equation (3). The dominant frequency of bispectrum estimate was then obtained by calculating the frequency that corresponded to the maximum value on the diagonal slice of the bispectrum estimate.

(2) $$\begin{array}{r} P\left(\omega \right)=\overset{N-1}{\underset{k\;=\;-N-1}{\sum }}R\left(k\right)e^{-j\omega k}=\frac{1}{N}|\overset{N-1}{\underset{l\;=\;0}{\sum }}x\left(n\right)e^{-j\omega l}{|}^{2} \end{array}$$

where *P*(ω) denotes the PSD estimate of the time sequence ${\left\{x\left(n\right)\right\}}_{n\;=\;0}^{N-1}$ with a finite amount *N*, and *R*(*k*) denotes the autocorrelation function of *x*(*n*).

(3) $$\begin{array}{r} \begin{array}{cc} B\left(\omega_{1},\omega_{2}\right) & =\overset{N-1}{\underset{k\;=\;-N-1}{\sum }}\overset{N-1}{\underset{l\;=\;-N-1}{\sum }}C\left(k,l\right)e^{-j\left(\omega_{1}k+\omega_{2}l\right)} \end{array} \end{array}$$

where *B*(ω_1_, ω_2_) denotes the bispectrum estimate of the time sequence ${\left\{x\left(n\right)\right\}}_{n\;=\;0}^{N-1}$ with a finite amount *N*, and *C*(*k, l*) denotes the third-order cumulant of ${\left\{x\left(n\right)\right\}}_{n\;=\;0}^{N-1}$.

In order to evaluate the effect of different factors, two methods were employed. The first compares the index of discrimination coefficient that gives a comprehensive consideration to both the mean value and the distribution of scalar separation (Equation 4)(Jakubowski et al., 2002). The results of the discrimination coefficient can range between 0 and positive infinity. Given that scalar A and scalar B are respectively the feature sequence of PD and of ET, a higher value of the discrimination coefficient indicates a larger difference between PD and ET. Based on pilot studies, the differentiation level of the index was set to be α > 0.7. If the calculated discrimination coefficient of the current condition is above the differentiation level, it indicates that current condition can give PD and ET a good differentiation.

(4) $$\begin{array}{r} \alpha_{A-B}\left(\varsigma \right)=|\frac{{\bar{x}}_{A}\left(\varsigma \right)-{\bar{x}}_{B}\left(\varsigma \right)}{\sigma_{A}\left(\varsigma \right)+\sigma_{B}\left(\varsigma \right)}| \end{array}$$

where α_*A*−*B*_(ς) denotes the discrimination coefficient of feature ς between scalar A and scalar B, ${\bar{x}}_{A}\left(\varsigma \right)$ and ${\bar{x}}_{B}\left(\varsigma \right)$ denote the mean values, and σ_*A*_(ς) and σ_*B*_(ς) denote the standard deviations.

The second method assesses the best separation between PD and ET with binary logistic regression Harrell (2015). Given the assumption of the binomial distribution, the possibility of each tremor instance was calculated as in Equation (5). Although the model is non-linear, it can be transformed into a linear model by taking logarithm, thus called a generalized linear model. The fitting process of the model was implemented with the generalized linear model regression function “glmfit” with the link of “logit” in Matlab. For ease of description, we assumed targeting the diagnosis of ET over PD in regression results. The threshold of the current feature for PD and ET was calculated as the cut-off value that maximized the Youden index (= TPR − FPR). The rationale behind this is to obtain the highest summation of sensitivity and specificity. With the best cut-off value, the optimal true positive rate (TPR, sensitivity), the optimal false positive rate (FPR, 1-specificity) and the best accuracy (ACC) would be calculated (Equations 6–8).

(5) $$\begin{array}{r} \pi_{i}=P\left(Y_{i}=1|X_{i}=x_{i}\right)=\frac{\exp\left(\beta_{0}+\beta_{1}x_{i}\right)}{1+\exp\left(\beta_{0}+\beta_{1}x_{i}\right)} \end{array}$$

where π_*i*_ denotes the possibility of instance *x*_*i*_ being true, and β_0_, β_1_ denote model parameters.

(6) $$\begin{array}{r} TPR=\frac{TP}{TP+FN} \end{array}$$

(7) $$\begin{array}{r} FPR=\frac{FP}{FP+TN} \end{array}$$

(8) $$\begin{array}{r} \text{ACC}=\frac{TP+TN}{TP+FP+FN+TN} \end{array}$$

To assess the overall performance of the feature, the receiver operating characteristic (ROC) analysis (Fawcett, 2006) was also performed based on the calculated possibilities of tremor instances. The index of area under the curve (AUC) was computed as in Equation (9). Higher AUC value indicates a better overall performance of the current feature ς. The index of AUC can range between 0 and 1.

(9) $$\begin{array}{r} AUC=\overset{\infty }{\underset{-\infty }{\int }}TPR\left(\varsigma \right)-FPR\left(\varsigma \right)d\varsigma \end{array}$$

where *AUC* denotes the area under the curve value of variable ς.

According to previous studies in assessing tremor, there was no concordant conclusion in tremor recording length, and the recording length of different studies could vary from seconds to several minutes. Although there have been studies investigating this aspect, such as the work by Di Biase et al. (2017), they were strongly restricted to some specific conditions or features of interest. In terms of the case in this research, such as without operator-dependent data screening, no research has targeted this before. To investigate the effect of tremor recording length here, we split the primitive tremor acceleration sequences with various time length, which were 5, 10, 20, 30, 60, 150, and 300 s, respectively. These time length were chosen based on the experience of previous studies and in order to take full advantage of the primitive length. The data were then processed and analyzed as aforementioned. In this part, we considered only the feature of dominant frequency and the differentiation between the overall PD cohort and ET cohort.

Statistics were finished with SPSS Version 22 (IBM, USA), with the basic level of statistical significance set at *p* < 0.05.

## 3. Results

All subjects completed the experiment without dropouts. A basic statistical analysis on the clinical features shows no significant difference between the PD cohort and ET cohort in terms of gender (*P* = 0.767), onset side (*P* = 0.783), and age [*t*_(48)_ = 0.464; *P* = 0.645].

### 3.1. Tremor intensity

**Figure 2:**
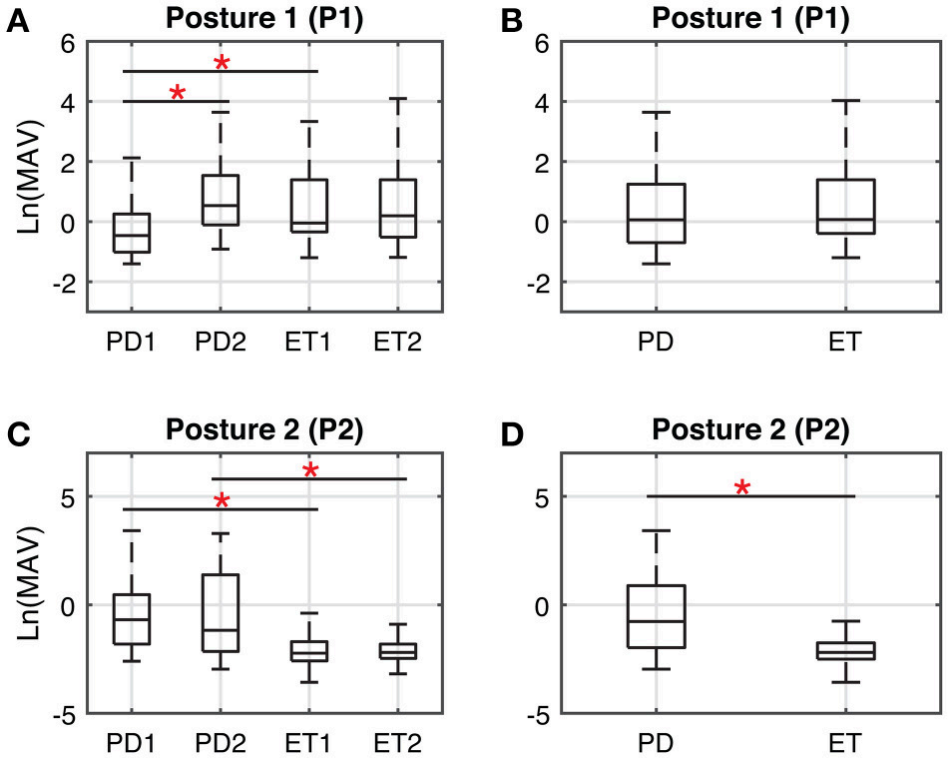
Boxplots representing the *Ln*(MAV) of the tremor acceleration signals. Horizontal lines of the box indicates maximum, upper quartile, median value, lower quartile and minimum of the group. Panels **(A,B)** illustrate the condition with the arm-stretching posture (P1); panels **(C,D)** illustrate the condition with the arm-rested posture (P2). In panels **(A,C)**, the group pairs considered for statistical analysis include: PD1-PD2, ET1-ET2, PD1-ET1, and PD2-ET2. In panels **(B,D)**, Group PD = PD1 + PD2; Group ET = ET1 + ET2. Significance level (^*^): *P* < 0.05.

Tests of Normality by the Shapiro-Wilk method showed that the data of MAVs failed to satisfy the requirement of a normal distribution. Thus, we normalized all MAVs by taking the natural logarithm. A two-way mixed-design ANOVA test was performed on ln (*MAV*) values with independent measures on group (PD, ET) and repeated measures on posture (P1, P2). Results showed there was a significant interaction between group and posture in ln (*MAV*) values [*F*_(1, 498)_ = 219.510, *P* < 0.05]. Simple effect analysis with independent-sample *t*-tests showed a significant difference between the PD cohort and the ET cohort with the arm-rested posture (P2) [*t*_(498)_ = 14.633, *P* < 0.05], while there was no significant difference between the PD cohort and the ET cohort with the arm-stretching posture (P1) [*t*_(498)_ = −1.813, *P* = 0.069]. To investigate the effect of stage, we performed a two-way ANOVA test with independent measures on both stage (S1, S2) and posture (P1, P2), in the PD cohort and in the ET cohort, respectively. In the PD cohort, a significant interaction between stage and posture was found [*F*_(1, 516)_ = 7.781, *P* < 0.05]. Further results of simple effect analysis showed there was a significant difference between the incipient stage (S1) and the progressed stage (S2) of PD with the arm-stretched posture (P1) [*t*_(258)_ = −5.226, *P* < 0.05] and meanwhile no significant difference between the two stages of PD with the arm-rested posture (P2) [*t*_(258)_ = −0.267, *P* = 0.790]. In the ET cohort, there were no significant interaction between stage and posture [*F*_(1, 476)_ = 1.430, *P* = 0.232] and no significant main effect of stage [*F*_(1, 476)_ = 0.762, *P* = 0.762]. Extra simple effect analysis between subgroups across PD and ET revealed a significant difference in group pairs: PD1-ET1 under P1 [*t*_(248)_ = −3.234, *P* < 0.05], PD1-ET1 under P2 [*t*_(248)_ = 10.762, *P* < 0.05] and PD2-ET2 under P2 [*t*_(248)_ = 9.451, *P* < 0.05] (Figure 2).

**Figure 3:**
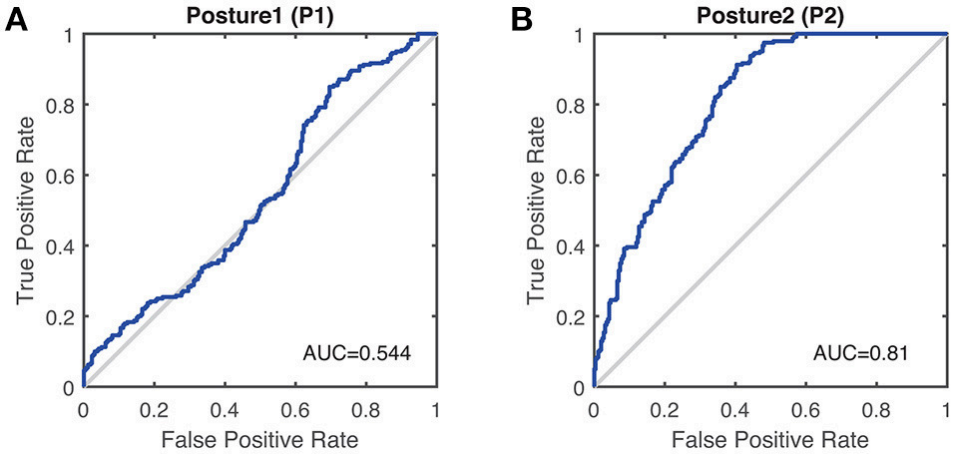
ROC curve of the *Ln*(*MAV*) as a performance measure to differentiate Parkinson's disease tremor and essential tremor, assuming targeting a diagnosis of essential tremor over Parkinson's disease. **(A)** With Posture 1 (P1). AUC is 0.544 (95%CI 0.493–0.595), with a standard error of 0.026. **(B)** With Posture 2 (P2). AUC is 0.810 (95%CI 0.773–0.847), with a standard error of 0.019.

Results of discrimination coefficient of *Ln*(*MAV*) in terms of different group pairs are shown in Table 2, where values above the differentiation level are presented in bold text. It is obvious that the discrimination coefficients of *Ln*(*MAV*) with the arm-rested posture (P2) were much larger than those with the arm-stretching posture (P1) and above the discrimination level of 0.70. Consistent results were found by the ROC analysis based on binary logistic regression by assuming targeting a diagnosis of essential tremor over Parkinson's disease (Figure 3). With the arm-stretching posture (P1), the optimal threshold of *Ln*(*MAV*) was found at −0.6 (TPR = 85%, FPR = 70%, ACC = 57%), while with the arm-rested posture (P2), the optimal threshold was at −1.2 (TPR = 91%, FPR = 40%, ACC = 75%). The index of AUC with the arm-rested posture (P2) was much larger than that with the arm-stretching posture (P1) (*AUC*_1_ = 0.544,*AUC*_2_ = 0.810).

**Table 2:** Discrimination coefficient (α) of *Ln*(MAV) in different group pairs: PD1-ET1, PD2-ET2, PD-ET.

		**Mean**	**SD**	**α**		**Mean**	**SD**	**α**		**Mean**	**SD**	**α**
P1	PD1	0.0	1.3	0.20	PD2	0.8	1.2	0.03	PD	0.4	1.3	0.08
	ET1	0.5	1.3		ET2	0.7	1.7		ET	0.6	1.5	
P2	PD1	0.4	1.5	**0.74**	PD2	−0.4	2.0	**0.71**	PD	−0.3	1.8	**0.72**
	ET1	−2.0	0.7		ET2	−2.1	0.5		ET	−2.1	0.6	

*Differentiation level: α≥0.70*.

*Values in the bold text indicate results above the differentiation level*.

### 3.2. Dominant frequency

**Figure 4:**
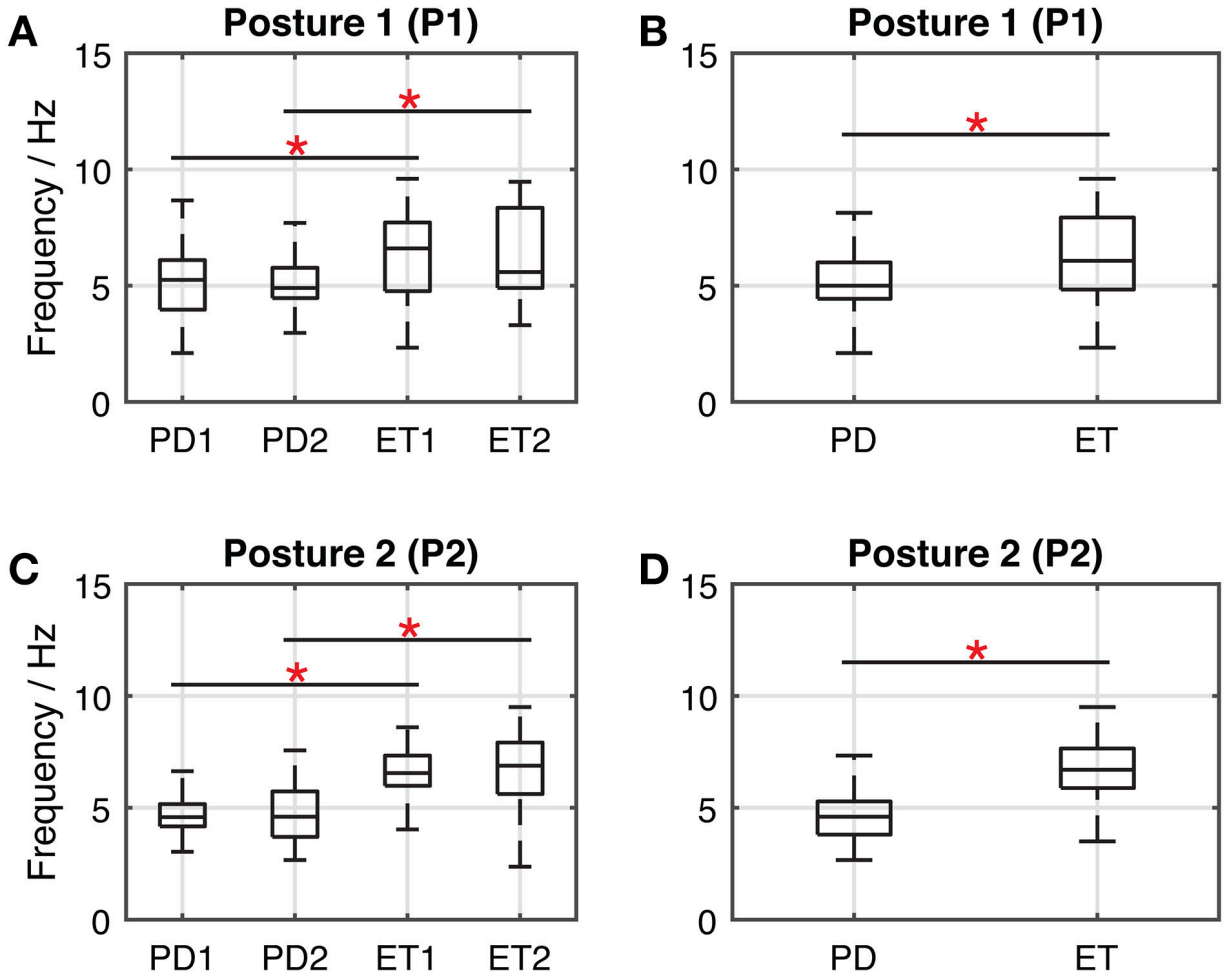
Boxplots representing the dominant frequencies (from the bispectrum analysis) of the tremor acceleration signals. Horizontal lines of the box indicates maximum, upper quartile, median value, lower quartile and minimum of the group. Panels **(A,B)** illustrate the condition with the arm-stretching posture (P1); Panels **(C,D)** illustrate the condition with the arm-rested posture (P2). In panels **(A,C)**, the group pairs considered for statistical analysis include: PD1-PD2, ET1-ET2, PD1-ET1, and PD2-ET2. Significance level (^*^): *P* < 0.05. Group PD = PD1 + PD2; Group ET = ET1 + ET2.

On the data of dominant frequency extracted from the bispectrum, a two-way mixed-design ANOVA test was performed [with independent measures on group (PD, ET) and repeated measures on posture (P1, P2)]. Results revealed there was a significant interaction between group and posture in dominant frequency [*F*_(1, 498)_ = 30.277, *P* < 0.05]. Simple effect analysis by independent-sample *t*-tests found a significant difference between the PD cohort and the ET cohort, with the arm-stretching posture (P1) [*t*_(498)_ = −7.311, *P* < 0.05] and with the arm-rested posture (P2) [*t*_(498)_ = −17.647, *P* < 0.05], respectively. The effect of the stage factor was investigated by performing a two-way ANOVA test with independent measures on both stage (S1, S2) and posture (P1, P2), in the PD cohort and in the ET cohort, respectively. In the PD cohort, no significant interaction between stage and posture [*F*_(1, 516)_ = 0.322, *P* = 0.570], and no significant main effect of stage [*F*_(1, 516)_ = 0.554, *P* = 0.457] were found. Similarly in the ET cohort, there were no significant interaction between stage and posture [*F*_(1, 476)_ = 0.272, *P* = 0.602], and no significant main effect of stage [*F*_(1, 476)_ = 0.802, *P* = 0.371]. Extra simple effect analysis between subgroups across PD and ET revealed a significant difference in the following group pairs: PD1-ET1 under P1 [*t*_(248)_ = −5.013, *P* < 0.05], PD2-ET2 under P1 [*t*_(248)_ = −5.461, *P* < 0.05], PD1-ET1 under P2 [*t*_(248)_ = −14.024, *P* < 0.05] and PD2-ET2 under P2 [*t*_(248)_ = −11.681, *P* < 0.05] (Figure 4). Statistical results of dominant frequency under the PSD estimate were omitted here because they were found to be highly similar to the above results of the bispectrum estimate.

**Figure 5:**
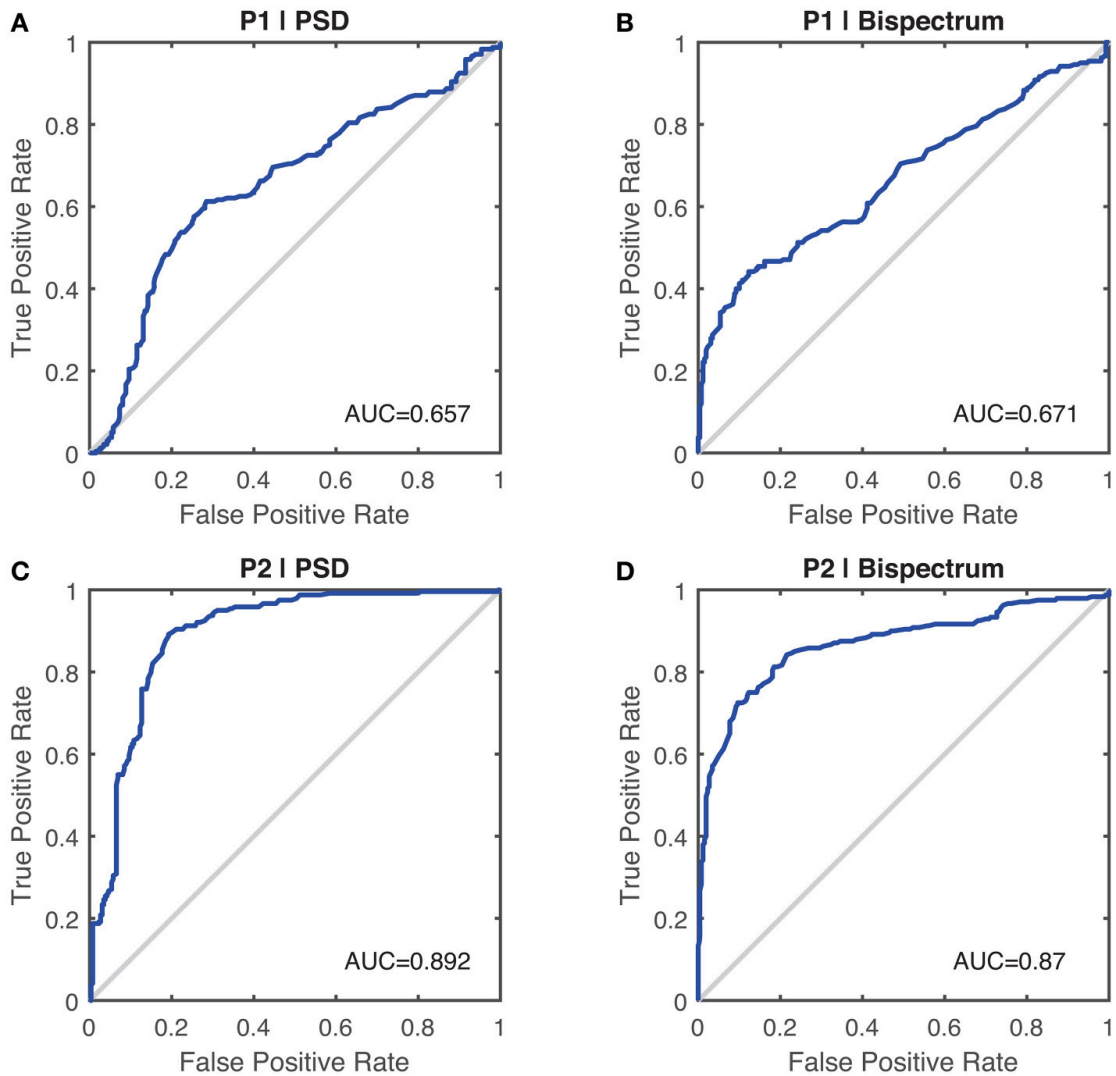
ROC curve of the dominant frequency as a performance measure to differentiate Parkinson's disease tremor and essential tremor, assuming targeting a diagnosis of essential tremor over Parkinson's disease. **(A)** Under the PSD estimate with Posture 1 (P1). AUC is 0.657 (95%CI 0.606–0.704) with a standard error of 0.025. **(B)** Under the bispectrum estimate with Posture 1 (P1). AUC is 0.671 (95%CI 0.621–0.716) with a standard error of 0.024. **(C)** Under the PSD estimate with Posture 2 (P2). AUC is 0.892 (95%CI 0.862–0.921) with a standard error of 0.015. **(D)** Under the bispectrum estimate with Posture 2 (P2). AUC is 0.870 (95%CI 0.835–0.902) with a standard error of 0.017.

**Figure 6:**
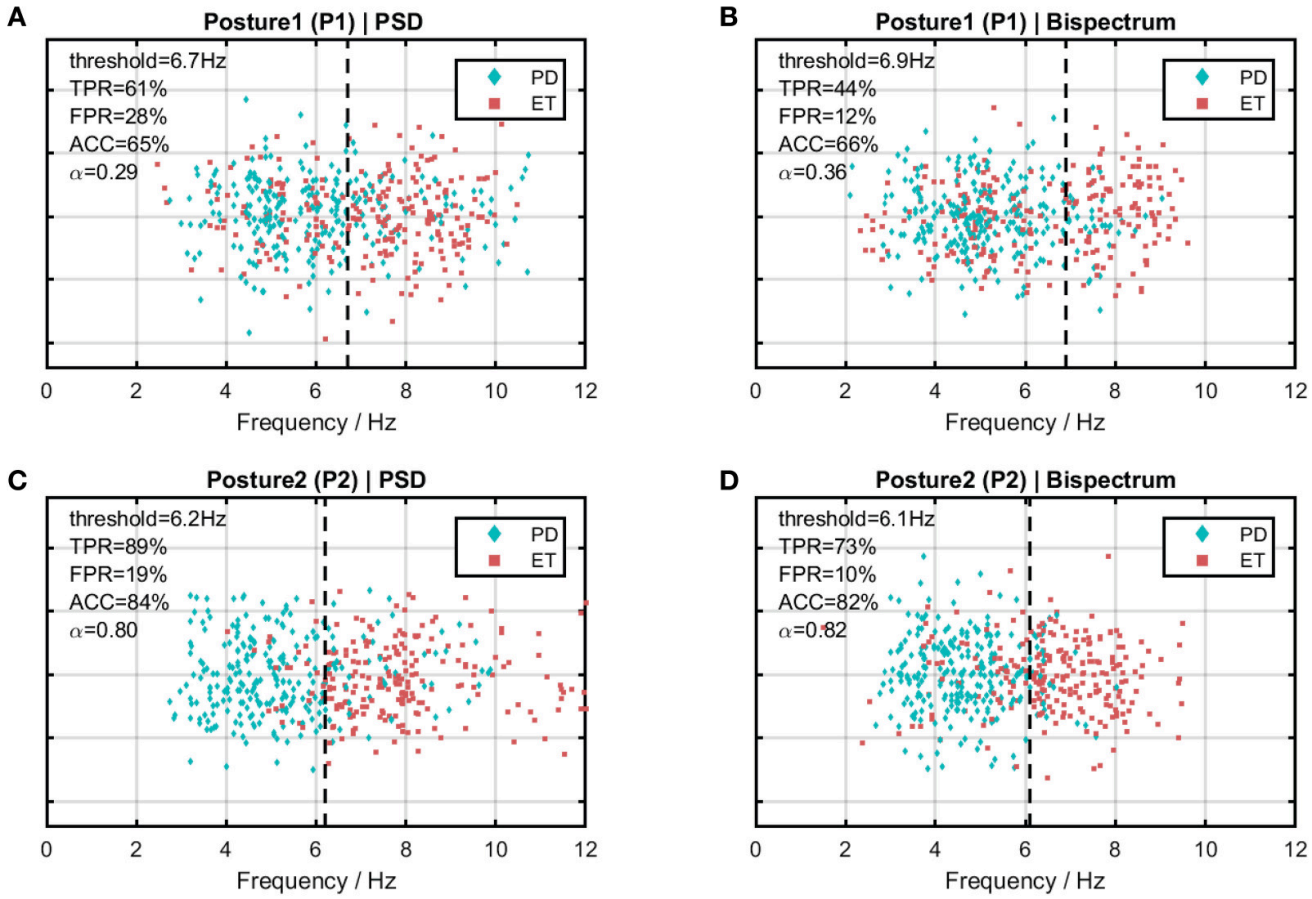
Scatter plots of dominant frequency distribution comparing the PD cohort and the ET cohort in different cases. Horizontal axis indicates frequency and the vertical dotted line indicates the optimal frequency threshold between the two cohorts. **(A)** Under the PSD estimate with Posture 1 (P1). **(B)** Under the bispectrum estimate with Posture 1 (P1). **(C)** Under the PSD estimate with Posture 2 (P2). **(D)** Under the bispectrum estimate with Posture 2 (P2).

Table 3 shows the computed discrimination coefficient of dominant frequency in terms of different group pairs. It could be noticed that the discrimination coefficients with the arm-rested posture (P2) were much larger than those with the arm-stretching posture (P1) and above the discrimination level of 0.70. In terms of the effect of the spectrum, discrimination coefficients yielded from the bispectrum estimate were slightly better than those from the PSD estimate, except for in the group pair of PD2-ET2 with P2. Results of ROC analysis based on binary logistic regression are shown in Figure 5. The optimal thresholds for separating the PD cohort and the ET cohort are shown in corresponding in Figure 6. As an example, the optimal threshold of the arm-stretching posture (P1) under bispectrum estimate was at 6.9 Hz (TPR = 44%, FPR = 12%, ACC = 66%), while that of the arm-rested posture (P2) under bispectrum estimate was at 6.1 Hz (TPR = 73%, FPR = 10%, ACC = 82%). The index of AUC with the arm-rested posture (P2) was found to be much larger than that with the arm-stretching posture (P1) under the PSD estimate (*AUC*_1_ = 0.657, *AUC*_2_ = 0.892) or under the bispectrum estimate (*AUC*_1_ = 0.671, *AUC*_2_ = 0.870), respectively.

**Table 3:** Discrimination coefficient (α) of dominant frequency in different group pairs: PD1-ET1, PD2-ET2, PD-ET.

**PSD**												
		**Mean**	**SD**	**α**		**Mean**	**SD**	**α**		**Mean**	**SD**	**α**
P1	PD1	6.2	1.7	0.22	PD2	5.9	1.8	0.30	PD	6.0	1.7	0.30
	ET1	6.9	1.7		ET2	7.0	1.9		ET	7.1	1.8	
P2	PD1	5.0	1.2	**0.86**	PD2	5.5	1.9	**0.80**	PD	5.2	1.6	**0.80**
	ET1	7.7	1.9		ET2	8.6	1.9		ET	8.1	2.0	
**BISPECTRUM**												
P1	PD1	5.2	1.9	0.32	PD2	5.2	1.3	0.35	PD	5.2	1.3	0.36
	ET1	6.3	1.9		ET2	6.3	1.8		ET	6.4	1.8	
P2	PD1	4.6	0.9	**0.90**	PD2	4.7	1.2	**0.74**	PD	4.7	1.0	**0.82**
	ET1	6.0	1.3		ET2	6.7	1.5		ET	6.7	1.4	

*Differentiation level: α≥0.70*.

*Values in the bold text indicate results above the differentiation level*.

### 3.3. Recording length

**Figure 7:**
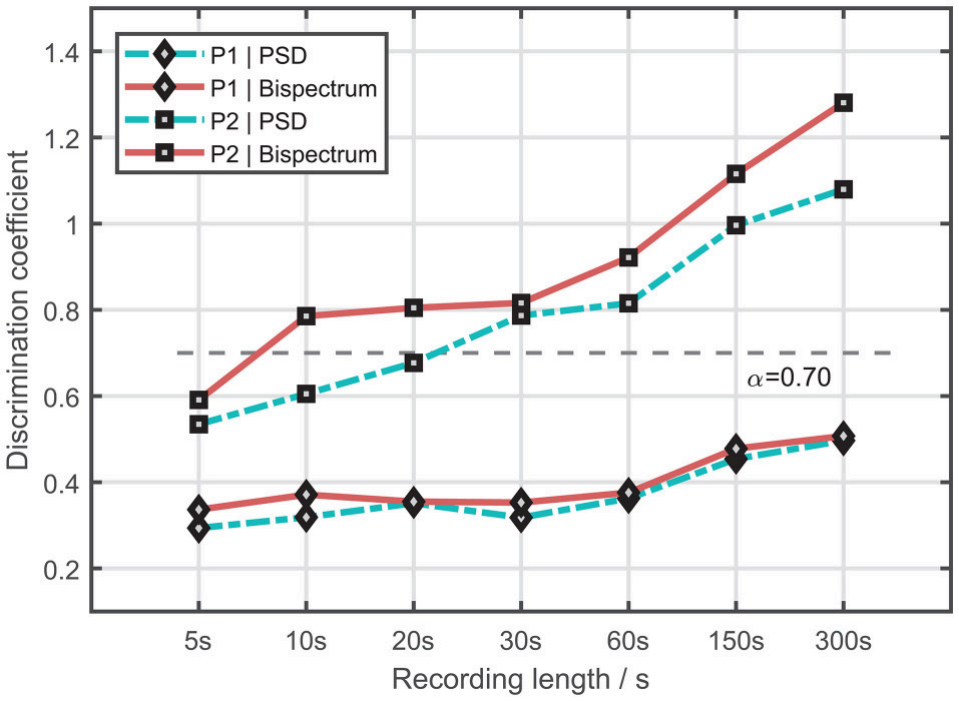
Discrimination coefficient (of dominant frequency) changes along various tremor recording length.

**Figure 8:**
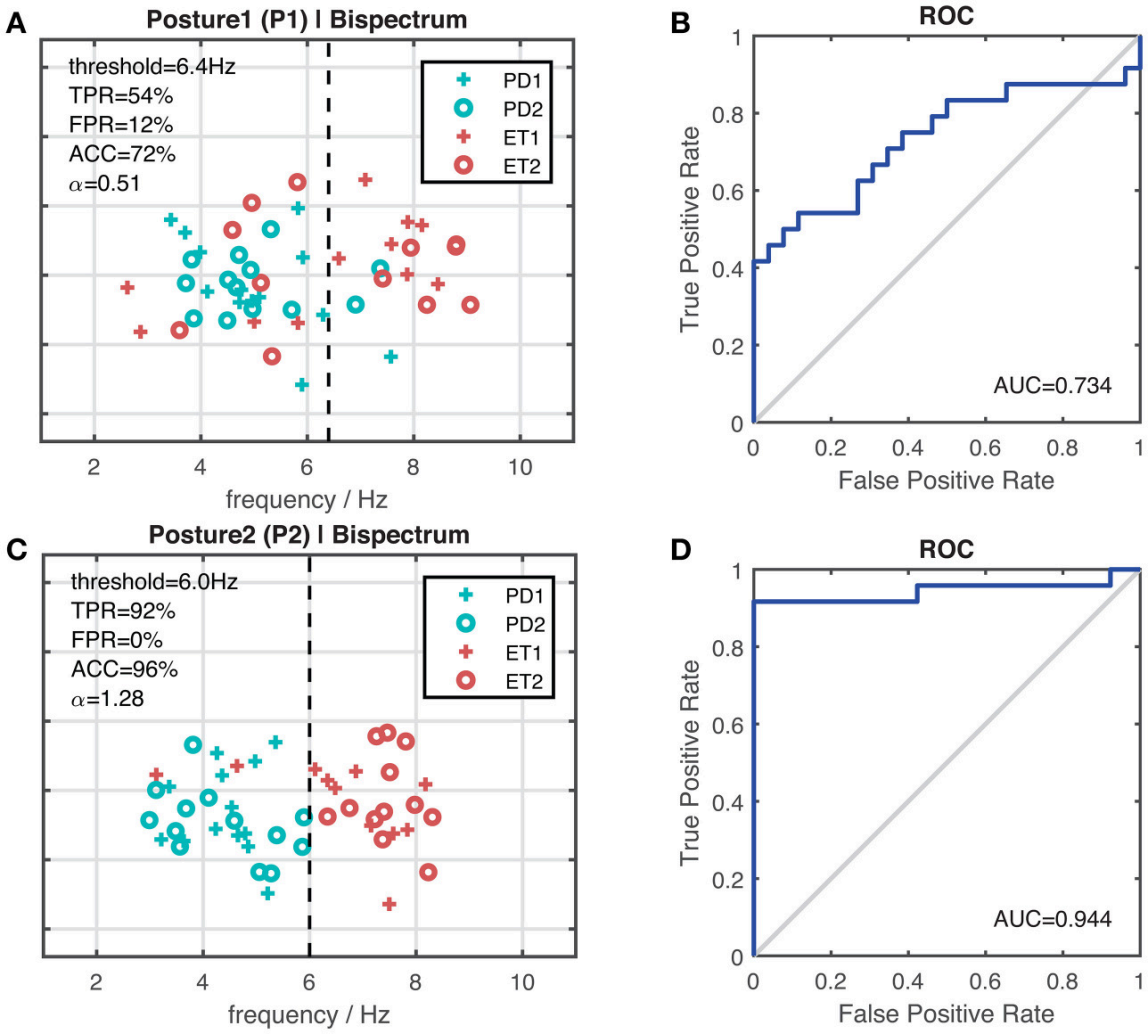
Results of dominant frequency with the maximum recording length of 300 s per segment in differentiating between the PD cohort and the ET cohort. **(A)** Scatter plot representing dominant frequencies under the bispectrum estimate with the arm-stretching posture (P1). **(B)** ROC curve corresponding to the case of **(A)**. AUC is 0.734 (95%CI 0.587–0.881) with a standard error of 0.075. **(C)** Scatter plot representing dominant frequencies under the bispectrum estimate with the arm-rested posture (P2). **(D)** ROC curve corresponding to the case of **(C)**. AUC is 0.944 (95%CI 0.863–1.000) with a standard error of 0.041.

Figure 7 shows the discrimination coefficient (of dominant frequency) changes along various recording length. Figure 8 presents the optimal result (in terms of discrimination coefficient) of the arm-stretching posture (P1) and the arm-rested posture (P2), respectively. For the arm-stretching posture (P1), the optimal threshold was at 6.4 Hz (TPR = 54%, FPR = 12%, ACC = 72%); and for the arm-rested posture (P2), it was at 6.0 Hz (TPR = 92%, FPR = 0%, ACC = 96%). ROC analysis showed the index of AUC was larger with the arm-rested posture (P2) than with the arm-stretching posture (P1) (*AUC*_1_ = 0.734, *AUC*_2_ = 0.944).

## 4. Discussion

Arm-stretching posture serves as the most common posture in eliciting the postural tremors that exist in the majority of tremor-affected patients. It reduces the difficulty of patient recruitment and standardizes the experimental protocol in tremor recording. In most diagnostic methods developed with an engineering perspective, this posture was applied. To employ this posture, the subject is required to stretch forward his/her arm and to keep the posture. In this case, many of his/her upper limb muscles and all of the joints will be involved. Given that stable moderate muscle loading can act as an add-on condition to ameliorate the arm-stretching posture in tremor differentiation (Burne et al., 2004), the arm-stretching posture may worsen tremor differentiation by introducing unstable muscle contraction and forces to the arm. In addition, redundant muscle activations may also exert extra proprioceptive feedback on the central nervous system, which in return may alter tremor stability (Jitkritsadakul et al., 2015).

Here, we propose the arm-rested posture, which was designed from two aspects: (1) removing redundant muscle activation and joint movements in the arm in order to avoid tremor pollution; (2) keeping forearm muscles and tendons passively strained in order to increase mechanical resonance. The results of the experiment demonstrate that the arm-rested posture is able to elicit trackable tremors in all tremor-affected patients including those without resting tremor. The absence of the resting tremor in those patients has been confirmed by monitoring the acceleration and sEMG signals while the subject rested in a recumbent posture. Therefore, the arm-rested posture differs distinctively from the resting posture which elicits only resting tremor.

In order to investigate the efficacy of the arm-rested posture, a two-session experiment was conducted on a cohort comprising 50 tremor-affected subjects. Note that the recruited subjects were heterogeneous in tremor manifestation, which served our aim to recruit a cohort representative of the vast majority of tremor-affected patients. The analysis on subjects' basic information excluded the significant effect of gender, of onset side, and of age, respectively.

In the aspect of tremor intensity, the significant difference between the PD cohort and the ET cohort was only found with the arm-rested posture. In the aspect of tremor frequency, although a significant difference between the PD cohort and the ET cohort was found with both the arm-stretching posture and the arm-rested posture, the significant interaction between group and posture indicated a better performance with the arm-rested posture. The results of assessment by the discrimination coefficient and the ROC analysis were in accordance with the results of statistical analysis. Therefore, in terms of the postures, we conclude that there is a better performance with the arm-rested posture than the arm-stretching posture in both tremor intensity and dominant frequency. As for the progress stage of the diseases, its significant effect was only found in tremor intensity within the PD cohort, indicating the fact that PD tremor deteriorates more significantly in amplitude than ET. As for the two spectrum estimates in calculating dominant frequency, the bispectrum estimate yielded slightly larger discrimination coefficients than the PSD estimate did, indicating the better anti-noise characteristics of the bispectrum estimate (Swami et al., 1998).

The factor of recording length was investigated on the aspect of dominant frequency. Results showed there was a rising trend in the index of discrimination coefficient as the recording length increased. Note that the horizontal axis is uneven in changes. The increasing rate of the index slowed down as the recording length increased by the same amount, approaching “ceiling”. Overall, the cases with the arm-rested posture yielded larger discrimination coefficients than those with the arm-stretching posture. The cases under the bispectrum estimate yielded slightly larger discrimination coefficients than those under the PSD estimate. The highest discrimination coefficient of each case was yielded at the maximum time length of 300s. The best result with the arm-rested posture was obtained under the bisepctrum estimate with the recording length of 300 s (AUC = 0.944, TPR = 92%, FPR = 0%, ACC = 96%), which was better than that with the arm-stretching posture in the same condition (AUC = 0.734, TPR = 54%, FPR = 12%, ACC = 72%). In comparison with the other electrophysiological measures identified for differentiation between Parkinson's disease tremor and essential tremor, we found that our best result with the arm-rested posture is superior to that of the state-of-the-art study by Di Biase et al. (2017), where the tremor stability index was proposed as a new tool for tremor differentiation (TPR = 95%, FPR = 5%, ACC = 92%). Although the sensitivity of our method was slightly lower, our method outperformed the tremor stability index in specificity and accuracy.

It is of interest to find that patients originally without resting tremor may exhibit trackable tremor under the arm-rested tremor although the tremor intensity is not high in the case. We assume that the elicited tremor may be a combination of postural tremor and resting tremor. While both of the arms are rested, the increased mechanical resonance in the arms by straining the forearm muscles and tendons may activated the proprioceptive feedback pathway regulated by the Golgi tendon organ, which senses changes in muscle tension and modulates the stretch reflex with afferent Ib fibers together (Prochazka and Gorassini, 1998; Helmich et al., 2013). As a result, patients who do not have resting tremor may generate postural components masquerading as resting.

The arm-rested posture may act as a universal tool to analyze tremor because it: (1) requires only acceleration measurement that is inexpensive and easy to access, (2) can be widely applied to patients with Parkinson's disease or essential tremor, and (3) may be assessed in a long term without muscle fatigue. In terms of the recorded tremor signal, there will be less polluted components than by the arm-stretching posture, and therefore a better differentiation between Parkinson's disease and essential tremor. Along with the methods proposed here, results demonstrate a complete separation between the two tremors with a recording length of 300 s (AUC = 0.944, TPR = 92%, FPR = 0%, ACC = 96%). In the overall process, no operator-dependent procedures, such as data screening, were employed. Therefore, the method is objective. In further research, it is important that we increase both the number and the types of tremor cases, such as the dystonic tremor and cerebellar tremor (Gövert and Deuschl, 2015), in order to fully investigate the efficacy of the arm-rested posture. In terms of limitation, the clinical evaluations of tremor by means of standardized clinical scales were not performed. Although their involvement would enhance the credibility of the results, they lack sufficient sensitivity and were thus not adopted.

## 5. Conclusion

In this paper, we propose a novel postural position, termed arm-rested tremor, for a better differentiation between Parkinson's disease and essential tremor. To investigate its efficacy, the posture was compared with another common posture, called arm-stretching posture, in fundamental aspects of tremor intensity and dominant frequency. The differentiation performance of the two postures were assessed by the index of discrimination coefficient and a receiver operating characteristic analysis based on binary logistic regression. The results of different assessments consistently demonstrate a better performance with the arm-rested posture than with the arm-stretching posture. We assume that it may act as a universal tool to analyze tremor for both clinical and research purpose.

## Author contributions

BZ contributed in carrying out the experiment, data analysis and drafting the manuscript. FH and LJ contributed in coordinating process of patient recruitment and participation. DZ conceived of the study and gave instructions on experimental design.

### Conflict of interest statement

The authors declare that the research was conducted in the absence of any commercial or financial relationships that could be construed as a potential conflict of interest. The reviewer AS and handling Editor declared their shared affiliation.
